# Role of Molecular Hydrogen in Ageing and Ageing-Related Diseases

**DOI:** 10.1155/2022/2249749

**Published:** 2022-03-18

**Authors:** Zhiling Fu, Jin Zhang, Yan Zhang

**Affiliations:** Department of Anesthesiology, Shengjing Hospital of China Medical University, Shenyang 110004, China

## Abstract

Ageing is a physiological process of progressive decline in the organism function over time. It affects every organ in the body and is a significant risk for chronic diseases. Molecular hydrogen has therapeutic and preventive effects on various organs. It has antioxidative properties as it directly neutralizes hydroxyl radicals and reduces peroxynitrite level. It also activates Nrf2 and HO-1, which regulate many antioxidant enzymes and proteasomes. Through its antioxidative effect, hydrogen maintains genomic stability, mitigates cellular senescence, and takes part in histone modification, telomere maintenance, and proteostasis. In addition, hydrogen may prevent inflammation and regulate the nutrient-sensing mTOR system, autophagy, apoptosis, and mitochondria, which are all factors related to ageing. Hydrogen can also be used for prevention and treatment of various ageing-related diseases, such as neurodegenerative disorders, cardiovascular disease, pulmonary disease, diabetes, and cancer. This paper reviews the basic research and recent application of hydrogen in order to support hydrogen use in medicine for ageing prevention and ageing-related disease therapy.

## 1. Introduction

**Figure 1:**
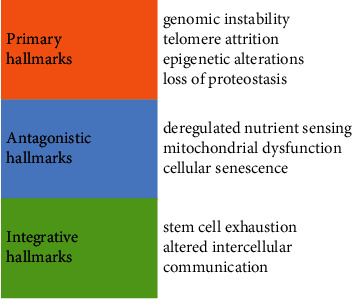
Hallmarks of ageing. Primary hallmarks are all considered to be unequivocally negative and cause cell damage. Antagonistic hallmarks exert beneficial effects at low levels but become harmful at high levels. Integrative hallmarks are results of previous two categories, directly influencing tissue homeostasis and function.

Ageing is a physiological process of progressive decline in an organism's functional reserve. It is almost universal throughout the living world [1]. Researchers have focused on exploring the underlying cellular mechanisms of ageing for decades [2] and have found that a variety of metabolic, biochemical, and molecular alterations that occur at a cellular level contribute to functional losses during the ageing process [3]. Nine candidate pathways contributing to the process of ageing have been identified and categorized as the “hallmarks of ageing” [4] (Figure 1).

Ageing represents a continuous risk of chronic noncommunicable diseases, such as neurodegenerative diseases, cardiovascular diseases (CVDs), diabetes, and cancer [5], although it is not the only factor. Over the past decades, the average human life expectancy has become substantially longer [6]. In particular, the absolute number of elderly people has increased in many countries [7]. Understanding the ageing mechanism and then further delaying the ageing process and the onset of age-related pathologies are of great importance.

Molecular hydrogen (H_2_) is a colorless, odorless gas and is the lightest among all gas molecules. Its therapeutic effect was first demonstrated in skin squamous carcinoma treatment [8]. In some bacteria, H_2_ can be enzymatically catabolized as an electron source. It can also be a product of anaerobic metabolism. In mammalian cells that have no functional hydrogenase genes, it was determined to be an inert gas that does not react with any biological compounds [9]. However, in 2007, investigators have discovered that H_2_ has antioxidant properties after selectively neutralizing hydroxyl radicals (•OH) and peroxynitrite (ONOO^−^) in cultured cells. It also prevented ischemia-reperfusion (I/R) injury and stroke in a rat model [10]. To date, prosurvival properties of some antioxidants have been demonstrated in some disease models [11]. H_2_ has been shown to improve lipid and glucose metabolism in patients with mild type 2 diabetes mellitus or impaired glucose tolerance [12]. Moreover, a recent study has shown that hydrogen-rich water (HRW) intake favorably affected several ageing-related features in healthy elderly, including extended mean telomere length, and tended to improve DNA methylation [13]. This review discusses the possible underlying mechanisms of H_2_ acting against ageing and its potential preventive and therapeutic applications in ageing-related diseases.

## 2. Potential Mechanisms of Molecular H_2_ Acting against Ageing

### 2.1. Antioxidation

#### 2.1.1. Oxidative Stress

Reactive oxygen species (ROS) and reactive nitrogen species (RNS) are reactive radical and nonradical derivatives of oxygen and nitrogen, respectively [14]. They are produced by all aerobic cells and play critical roles in both normal physiological and pathological conditions. ROS and RNS are generated through endogenous and exogenous routes. Endogenous routes include ROS generated in mitochondria nicotinamide adenine dinucleotide phosphate (NADPH) oxidase, lipoxygenase, and angiotensin II. Exogenous routes include air and water pollution, tobacco, alcohol, heavy metals, industrial solvents, cooking, and radiation, which are metabolized into free radicals inside the body [14, 15].

The oxidative stress occurs when there is an imbalance in formation and removal of ROS and RNS due to metabolic and pathophysiological changes and environmental stress exposure [16].Oxidative stress can cause accumulative oxidative damage in macromolecules (lipids, DNA, and proteins) and eventually lead to age-associated functional losses [14, 17, 18]. Genomic instability is a common denominator of ageing. *In vitro* studies have shown that ROS can induce DNA damage by directly oxidizing nucleoside bases and inducing replication stress [19]. They also cause mitochondrial DNA (mtDNA) strand breaks and degradation [20] *in vivo*, while ionization radiation and ultraviolet light exposure may also be associated with DNA damage. However, it may not be a key species participating in endogenous oxidative DNA damage [21]. Moreover, researchers in recent years have unexpectedly observed that increasing ROS does not accelerate ageing, while decreasing ROS levels by increasing antioxidant defenses may result in shortened lifespan [17]. Nevertheless, ROS and RNS may play a critical role in the ageing process, and the relationship between ROS/RNS and ageing is complex. ROS and RNS can both be beneficial and detrimental depending on the species and conditions.

#### 2.1.2. Characteristics of Antioxidative Effect due to H_2_

The antioxidant activity of H_2_ is the basis of its preventive and therapeutic effects. H_2_ has been shown to exert its beneficial effects in various pathological conditions that involve free radicals and oxidative stress [22–24], as reflected by a reduction in malondialdehyde (MDA), 8-hydroxy-2′-deoxyguanosine (8-OHdG), myeloperoxidase (MPO), and 4-hydroxynonenal (4-HNE).

**Figure 2:**
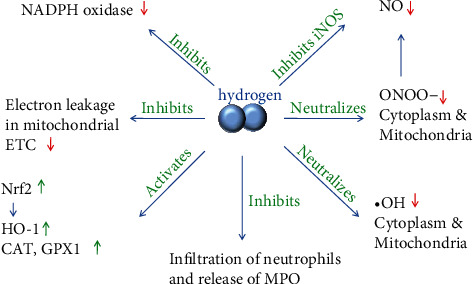
Antioxidative effect of H_2_. H_2_ can directly neutralize •OH and ONOO^−^, reduce NO production by inhibiting iNOS expression and eliminating NO-derived ONOO^−^ while suppressing NADPH oxidase and MDA, and decrease ROS in mitochondria, which is the main ROS generation location. In addition, H_2_ can activate Nrf2, inducing HO-1 expression and enhancing the transcription of CAT, GPX1, and GSH.

The mechanism of antioxidative effect due to H_2_ involves the following aspects (Figure 2):

*(1) H_2_ Directly Neutralizes •OH*. The •OH is produced by the Fenton reaction and Haber-Weiss reaction [25, 26], and •OH formed *in vivo* reacts with biomolecules present at its formation site, making it difficult to trap •OH and directly demonstrate its formation in the biological systems [25]. H_2_ can accumulate in the lipid phase more than in the aqueous phase, especially in the unsaturated lipid region, which is the main location for the primary free radical chain reactions [27]. Therefore, H_2_ may have an advantage in suppressing these reactions.

*(2) H_2_ Directly Scavenges ONOO^−^*. Compared to •OH, the half-life of ONOO^−^ is long, which has a greater chance to react with H_2_ at the lesion site [28, 29]. In addition, H_2_ inhibits the generation of nitrotyrosine, which reflects the generation of ONOO^−^ [30]. However, there is a controversy regarding the direct reaction of H_2_ with ONOO^−^ and its influence on tyrosine nitration by ONOOH [31]. This discrepancy may be caused by different experimental conditions and investigators and requires further study.

*(3) H_2_ Indirectly Reduces Nitric Oxide (NO) Production*. NO is produced by nitric oxide synthase (NOS). High amounts of NO resulting from inducible NOS (iNOS) can trigger the inflammatory process, which is associated with ageing and inflammatory conditions, such as type 2 diabetes and Alzheimer's disease (AD) [32]. H_2_ does not scavenge NO. However, it inhibits iNOS expression [33, 34], decreasing its related NO production. Additionally, H_2_ may eliminate the NO-derived ONOO^−^, which is formed through a reaction between superoxide anion (O_2_^•^) and NO. This may consume NO and indirectly decrease its quantity [35].

*(4) H_2_ Inhibits NADPH Oxidase Activity*. NADPH oxidase is a prooxidative enzyme that transfers electrons from NADPH to oxygen to generate O_2_^•^ and other downstream ROS [36]. Several homologs of the cytochrome NADPH oxidase subunit have been found, including NOX1-5, DUOX1, and DUOX2 [36]. H_2_ suppresses the NADPH oxidase activity and downregulates NOX2 and NOX4 expression, which are notably relevant to cardiac pathophysiology, such as cardiac hypertrophy and interstitial fibrosis [37, 38]. Further study has shown that H_2_ decreased the levels of NADPH oxidase subunits, including p40 phox, p47 phox, and p67 phox in the cell membrane, but increased their levels in the cytoplasm. By limiting the translocation of these molecules to the cell membrane, H_2_ reduces the NADPH oxidase activity [39].

*(5) H_2_ Decreases Mitochondrial ROS*. ROS are mainly generated in the mitochondria [40]. H_2_ is the smallest molecule and therefore capable of passing through the mitochondrial membrane to neutralize •OH and ONOO^−^ [41]. In addition, H_2_ suppresses electron leakage in the electron transport chain (ETC), prevents superoxide generation in the mitochondrial complex I, rectifies the electron flow, and thus suppresses oxidative damage in the mitochondria [42].

*(6) H_2_ Induces Antioxidant Gene Expression and Increases Antioxidant Enzyme Activity*. In addition to directly reducing oxidative stress, H_2_ can trigger the antioxidation systems. The NF-E2-related factor 2 (Nrf2) functions as an important defense system against oxidative stress by inducing expression of various genes, such as heme oxygenase1 (HO-1). H_2_ can activate Nrf2 and induce its translocation into the nucleus, enhancing the transcription of catalase (CAT) and glutathione 1 (GPX1) [43].

*(7) Neutrophil Activity Action*. Neutrophils are great producers of ROS and play a role in ageing [44]. H_2_ reduces neutrophil infiltration in the injured tissue [45], potentially decreasing the generation of ROS. MPO is a heme-containing peroxidase that is mainly expressed in neutrophils. It plays an important role in microbial killing by neutrophils but is also a local mediator of tissue damage and the resulting inflammation in various inflammatory diseases [46]. As discussed above, H_2_ can decrease the amount of MPO [47], which may be associated with inhibition of its release by neutrophils.

#### 2.1.3. Impact of H_2_ on Ageing Hallmarks via antioxidative Effect

*(1) Maintaining Genome Stability*. As mentioned above, ROS contribute to accumulative DNA damage, which is one of the common denominators of ageing. H_2_ protects against DNA damage caused by various stimulations through its antioxidative effect. In radiation-caused DNA damage, H_2_ alleviated nucleobase DNA damage in aerated aqueous solutions [48] and reversed exhausted cellular endogenous antioxidants [49]. In ultraviolet A- (UVA-) induced skin damage, H_2_ significantly alleviated nuclear condensation and DNA fragmentation of keratinocytes [50]. Similarly, in cigarette smoke- (CS-) induced emphysema, H_2_ significantly decreased phosphorylated histone H2AX and 8-OHdG levels, which are markers of oxidative DNA damage [51]. Oral administration of water containing hydrogen-rich saline (HRS) prepared in alternating current electrolysis was effective for preventing systemic oxidative DNA injuries and for clinical diabetes treatment [52]. These findings suggest that H_2_ can potentially intervene in accumulation of genetic damage in the living body caused by oxidative stress and alleviate the ageing process.

*(2) Modulating Cellular Senescence*. Cellular senescence is a stress response characterized by arrested cell proliferation and resistance to apoptosis [53]. It takes part in tumor suppression and ageing process in vertebrates and plays an important role in maintaining body homeostasis [54, 55]. However, senescent cells are also drivers of ageing that contribute to a series of age-related pathologies [55].

H_2_ modulates cell senescence in multiple cell types. When human umbilical vein endothelial cells were induced by 2,3,7,8-tetrachlorodibenzo-p-dioxin, which can strongly induce cellular senescence, the cells exhibited increased expression of 8-OHdG and acetyl-p53, decreased the ratio of NAD (+) to NADPH, impaired Sirt1 activity, and activated senescence-associated protein *β*-galactosidase. H_2_ inhibited these senescence-related changes by activating the Nrf2 pathway [56]. When H_2_ was produced in nanoparticles that do not easily disappear and collapse after a long period of time under water, it inhibited the accumulation of *β*-galactosidase in hydroxyurea-induced oxidative stress and protected against senescence and death in murine embryonic fibroblasts [57]. In a pyocyanin-stimulated cyto •OH-induced cellular senescence model, supersaturated concentrations of H_2_ added into the cell culture medium suppressed cyto •OH-mediated lipid peroxide formation and cellular senescence induction, and the investigator speculated that H_2_ generated in human gut bacteria may be involved in the suppression of aging [58].

*(3) Effect on Epigenetic Alterations*. Epigenetic alterations include alterations in modification of histones, DNA methylation, and chromatin remodeling [4].

For histone modification, manipulations of histone-modifying enzymes may influence the ageing process [4]. Studies have shown that H_2_ can modulate histone methylation and acetylation.

In the liver of mice and rats, H_2_ treatment changed the H3K27 methylation status and induced H3K27 demethylase, which can activate mitochondrial unfolded protein response-related genes to protect the mitochondrial function. It also activated the expression of a series of genes regulated by the histone H3K27 methylation status [59].

Sirtuins are NAD (+)-dependent histone deacetylases that regulate various physiological functions. Human sirtuin isoform Sirt1-7 is considered an attractive therapeutic target for aging-related diseases [60]. Studies have shown that H_2_ can modulate the sirtuin family via its antioxidative effect. In the kidneys, H_2_ suppressed the downregulated Sirt3 expression, which is the most abundant member of the sirtuin family, by reducing oxidative stress reactions [61]. In the liver, H_2_ elevated HO-1 to induce Sirt1 expression, inhibited the inflammatory response and apoptosis, and suppressed palmitate-mediated abnormal fat metabolism [62, 63]. In the blood vessels, H_2_ inhibited oxidized low-density lipoprotein and induced inflammatory cytokine expression via Sirt1-mediated autophagy, potentially inhibiting the progression of atherosclerosis [64].

The effects of H_2_ on DNA methylation and chromatin remodeling remain unclear.

*(4) Effect on Telomere Attrition*. Telomeres are particularly susceptible to age-related deterioration. Physically, ageing in mammals is accompanied by a progressive loss of telomere length and function due to normal replication [65, 66]. The telomere shortening rate may be accelerated by oxidative stress [67]. It can thus be inferred that H_2_ can alleviate telomere shortening via its action on inflammation and oxidative stress. However, studies that have specifically explored the effect of H_2_ on telomere maintenance are limited. Recently, a randomized controlled pilot trial showed that HRW intake for six months extended mean telomere length by ~4% [13]. More studies are still needed to determine the intervention effect of H_2_ on telomere-lengthening and to identify its potential mechanism.

Collectively, these multiple lines of inquiry indicate that by modulating ROS and reducing oxidative stress, H_2_ holds a great promise to maintain DNA stability, modulate cell senescence, alleviate epigenetic alterations and telomere attrition, and extend a healthy lifespan [68].

### 2.2. Anti-inflammation

#### 2.2.1. Inflammation and Inflamm-Ageing

Inflammation is a protective life process that repairs damaged lesions and restores homeostasis by inhibiting injurious activators. It is a dynamic and continuous remodeling network as a result of the interaction among genes, lifestyles, and environments [69, 70]. However, it is not always helpful and may even be harmful when it persists and becomes chronic [71, 72]. It is now increasingly recognized that inflammation is the common molecular pathway that underlies the pathogenesis of diverse diseases ranging from infection to chronic ageing-related diseases and ageing itself [73]. The so-called “inflamm-ageing” is a chronic subclinical systemic progressive increase in inflammation and is an important characteristic of the ageing process [74]. The extended lifespan may be a consequence of pro- and anti-inflammatory process fine-tuning [75]. Thus, imbalance in pro- and anti-inflammatory cytokines may take part in the process of inflamm-ageing. In addition, imbalance in age-related redox, DNA damage, decreased autophagy activity, and increased senescent cell numbers, especially in the immune system with ageing, also play important roles in the process of inflamm-ageing [72, 76].

#### 2.2.2. Anti-inflammatory Effect of H_2_ and Its Impact on Ageing Hallmarks

The mechanism for the anti-inflammatory effects of H_2_ involves several aspects.

1. H_2_ reduces the release of proinflammatory cytokines, including interleukin- (IL-) 1*β*, IL-6, tumor necrosis factor-*α* (TNF-*α*), nuclear factor kappa B (NF-*κ*B), and high-mobility group box 1 (HMGB1) [77–79]. It also increases the level of anti-inflammatory cytokines, such as IL-4, IL-10, and IL-13 [63, 80]
2. H_2_ promotes macrophage polarization from proinflammatory M1 type to anti-inflammatory M2 type, which in turn generates additional anti-inflammatory cytokines, such as IL-10 and transforming growth factor- (TGF-) *β* [80]
3. H_2_ reduces the aggregation and infiltration of macrophages and neutrophils [81, 82]
4. The anti-inflammatory effect of H_2_ may involve inhibiting several inflammatory pathways. (1) NF-*κ*B pathway: H_2_ inhibits the NF-*κ*B pathway in various disease conditions. It is the most common inflammatory pathway that takes part in a variety of pathological models, including the ageing process [67, 83]. (2) NLRP3 pathway: H_2_ inhibits NLRP3, which fuels both chronic and acute inflammation and contributes to inflamm-ageing [84, 85]. (3) Toll-like receptor (TLR) 4-mediated inflammatory pathway: H_2_ inhibits TLR4, which involves hyperglycemia in type 2 diabetes mellitus [86]

Inflammation is a prominent ageing-related process that alters intercellular communication. H_2_ also inhibits chronic inflammation, which may contribute to inflamm-ageing. For example, it improved inflammation biomarkers in patients with metabolic syndrome [87] and attenuated inflammatory airway status in patients with asthma and chronic obstructive pulmonary disease (COPD), especially tobacco smoke-induced COPD [88]. In the brain, H_2_ can inhibit neuroinflammation caused by a variety of pathological conditions, such as cerebrovascular disease, neonatal brain disorders, and neurodegenerative disease [89]. Therefore, H_2_ can effectively attenuate the inflammation process in diverse pathological conditions, slow down the inflamm-ageing process, and prevent ageing-related diseases. Further studies are needed to investigate how H_2_ regulates the physiological process of ageing via its anti-inflammatory effects.

### 2.3. Regulating mTOR and Autophagy

#### 2.3.1. mTOR, Autophagy, and Ageing

mTOR is a multifunction protein that can integrate signals based on nutrient availability, energy status, growth factors, and various stressors and regulate key cellular processes, including mRNA translation, protein synthesis, autophagy, transcription, and mitochondrial function. All of these functions are involved in maintaining cellular homeostasis and modulating extended lifespan [90, 91]. Therefore, mTOR is a key modulator of ageing and age-related disease [92].

Autophagy is an evolutionarily ancient and highly conserved catabolic process that involves a series of evolutionarily conserved autophagy-related genes (Atg) [93, 94]. mTOR is a primordial negative modulator of human autophagy and is inhibited under fasting conditions by activating mTOR targets ULK1, ULK2, and Atg13 [95]. A previous study has shown that increased autophagy delayed ageing and extended longevity while decreasing autophagy by mutating essential Atg genes that inhibit longevity [96].

#### 2.3.2. Modulatory Effect of H_2_ on mTOR and Autophagy and Its Impact on Ageing Hallmarks

Deregulated nutrient-sensing and loss of proteostasis are two other ageing hallmarks. mTOR belongs to one of the nutrient-sensing systems. Dysregulation of mTOR signaling can result in metabolic disorders, neurodegeneration, cancer, and ageing [97]. For example, the activity of mTOR increases during ageing and contributes to age-related obesity. This can be reversed by directly infusing rapamycin to the hypothalamus [98]. Impaired proteostasis, such as misfolded or aggregated proteins, contributes to the development of AD, Parkinson's disease (PD), and cataracts. Proteostasis is maintained by stabilizing correctly folded proteins and by degrading proteins through the proteasome or lysosome [4, 99]. The autophagy-lysosomal system often experiences an ageing-associated decline [100]. Therefore, measurements targeting autophagy can potentially improve proteostasis and delay the ageing process.

H_2_ modulates mTOR and autophagy in multiple diseases and conditions. For example, H_2_ inhibits mTOR, activates autophagy, and alleviates cognitive impairment resulting from sepsis [101]. It inhibits the activation of the PTEN/AKT/mTOR pathway and alleviates peritoneal fibrosis [102]. The activated mTOR/TFEB autophagy alleviates the LPS-induced endothelial damage [103].

It also facilitates autophagy-mediated NLRP3 inflammasome inactivation and alleviates mitochondrial dysfunction and organ damage [104, 105]. In chronic diseases, H_2_ activates FoxO1-mediated autophagy and exerts beneficial effects on chronic cerebral hypoperfusion-induced cognitive impairment [106].

Most of the studies have focused on the pathological conditions. At present, there is no direct evidence that H_2_ administration delays the normal ageing process through autophagy. However, it is conceivable that long-term administration of H_2_ can modulate mTOR and autophagy to help remove aggregated or misfolded proteins or defective organelles, subsequently maintaining proteostasis and cellular homeostasis and potentially delaying the ageing process and ageing-related diseases.

Paradoxically, H_2_ may inhibit autophagy in some conditions [107].

Autophagy is a two-edged sword, as its excess may cause cell death and have other harmful effects on the body. Nonetheless, H_2_ can harness autophagy to achieve the ultimate goal of maintaining homeostasis in the body.

### 2.4. Regulating Mitochondria

#### 2.4.1. Mitochondria and Ageing

Mitochondria are cellular powerhouses for producing ATP required by the cell [108]. In addition, emerging investigations have focused on their role in ageing. As cells and organisms age, the efficacy of the respiratory chain tends to decrease, leading to an increase in electron leakage and a reduction in ATP generation [109]. The mechanisms involved in mitochondrial ageing include mtDNA damage, oxidation of mitochondrial protein, dysregulation of mitochondrial dynamics, and impaired mitophagy that causes the accumulation of aberrant mitochondria as demonstrated in cardiovascular, metabolic, and neurodegenerative disorders [110–113].Therefore, mitochondria are promising therapeutic targets for influencing specific age-related disorders [111].

#### 2.4.2. Protective Effect of H_2_ on Mitochondria and Its Impact on Ageing Hallmarks

Mitochondrial dysfunction is one of the ageing hallmarks. Improving mitochondrial function may delay the ageing process and extend lifespan.

As mentioned above, H_2_ prevents mitochondrial oxidative stress by directly neutralizing ROS in mitochondria and suppresses the electron leakage in ETC. In addition, H_2_ can improve mitochondrial function represented by the following mechanism: (1) H_2_ can block the opening of the mitochondrial permeability transition pores and restore mitochondrial construction and function in the cell [114]; (2) H_2_ regulates mitochondrial dynamics by increasing the levels of MFN2 and decreasing Drp1 [115]; (3) H_2_ modulates mitophagy, which is an important mitochondrial quality control mechanism, and alleviates inflammation and apoptosis in tissue injury [116, 117]; (4) H_2_ can target mitochondria to improve the energy metabolism. It stimulates mitochondrial ETC function and increased levels of ATP production by complex I and II substrates [118]. (5) H_2_ modulates mitohormesis, a process in which low and noncytotoxic concentrations of ROS promote mitochondrial homeostasis [119], as manifested by enhanced mitochondrial activities with an elevated level of oxidative stress, and then increases expression of antioxidative enzymes [43].

These findings outline the possibilities that H_2_ targets mitochondria to prevent ageing-related injury, providing a new way to delay ageing and ageing-related disorders.

### 2.5. Regulating Apoptosis

#### 2.5.1. Apoptosis and Ageing

Apoptosis is a canonical form of programmed cell death [120]. It plays an indispensable role in both physiological and pathological conditions. For example, it is involved in developmental processes, including cell differentiation and tissue remodeling, it provides an important anticancer mechanism, and the p53 pathway is a vital modulator in this response [121]. Abnormal regulation of apoptosis is associated with a variety of human diseases, including developmental disorders, neurodegeneration, and cancer [122]. Ageing is associated with decreased apoptosis and increased cell senescence. Increased resistance to apoptosis in the ageing process can lead to the survival of postmitotic cells but at the price of damaging housekeeping functions [123].

#### 2.5.2. Effect of H_2_ on Apoptosis and Its Impact on Ageing Hallmarks

H_2_ can modulate apoptosis in various disease models. In most cases, H_2_ protects tissue from injury through antiapoptotic effects, such as inhibiting the expression of proapoptotic factors Bax, caspase-3, caspase-8, and caspase-12, inhibiting p53 signaling, and upregulating antiapoptotic factors, such as Bcl-2 and Bcl-xl [124–126]. However, it may promote apoptosis in some conditions. For example, apoptosis evasion is a prominent hallmark of cancer that is closely associated with ageing, where H_2_ increases rates of early and late apoptosis in lung cancer [127, 128], facilitates scavenging of carcinoma cells in the body, and reduces proliferation of cancer cells. This proapoptotic effect in cancer cells indicates that H_2_ can modulate cell death to protect the body against harmful attacks and maintain homeostasis in the body. Whether H_2_ can affect ageing hallmarks through apoptosis remains unknown and requires further studies.

**Figure 3:**
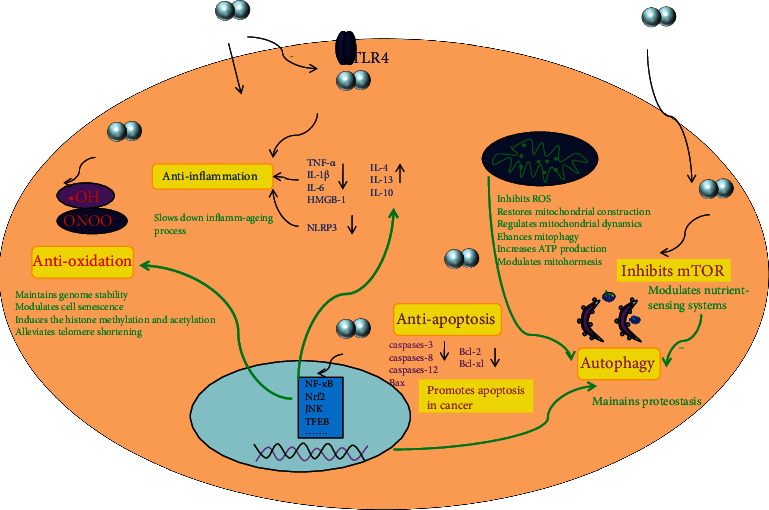
Potential mechanisms for H_2_ action against ageing and the influence on ageing hallmarks, including antioxidative stress, anti-inflammation, mTOR regulation, autophagy, apoptosis, and mitochondria.

The antiageing mechanism of H_2_ and the influence on ageing hallmarks are summarized in Figure 3.

## 3. Prevention and Therapy Using H_2_ in Ageing-Related Diseases

As many infectious diseases can be cured, more and more people now die of noncommunicative diseases, although these types of illnesses cannot be simply attributed to ageing alone. Efforts to delay the onset of diseases have been made in the past decades, but most diseases still maintain a significant impact on the population [129]. The studies on H_2_ in the areas of prevention and therapy in ageing-related diseases may provide some information for treating these conditions in human beings.

### 3.1. Effects of H_2_ on Neurodegenerative Disorders

#### 3.1.1. Effects of H_2_ on AD

In AD, A*β* accumulation stimulates a proinflammatory response in resident immune cells, microglia, and astrocytes in the brain, leading to plaque phagocytosis, as well as their proteolytic degradation. In addition, the aggravated proinflammatory state occurring during the process of disease can trigger the hyperphosphorylation of tau [130]. Furthermore, microglia, which produce excessive A*β* and become senescent in the progression of AD, continue to produce proinflammatory, microglia-recruiting mediators, including cytokines and chemokines. This results in them becoming overactive in neurodegeneration, eventually leading to more microglia becoming senescent [131].

Animal studies have shown that H_2_ can alleviate AD by inhibiting the inflammatory response and oxidative stress. In a rat model utilizing intracerebroventricular injection of A*β*, intracerebroventricular injection of hydrogen saline (HS) prevented A*β*-induced neuroinflammation and oxidative stress, significantly suppressed inflammatory cytokines (IL-6, TNF-*α*, and IL-1*β*), MDA, and 8-OHdG, and improved memory dysfunction [132]. A further study has demonstrated that H_2_ attenuates the activation of c-Jun NH₂-terminal kinase (JNK) and nuclear NF-*κ*B, which are involved in neuroinjury [133]. HRW can also upregulate Sirt1-Forkhead box protein O3a (FOXO3a) by stimulating AMP-activated protein kinase to alleviate potential A*β*-induced mitochondrial loss and oxidative stress [134]. In addition to suppressing memory impairment and neurodegeneration, drinking hydrogen water (HW) directly extended the mean lifespan in a dementia rat model. Interestingly, in a transgenic AD mouse model, investigators found that three months of HRW treatment more profoundly ameliorated oxidative stress and inflammatory responses in the brains of female transgenic AD mice than in those of males. This sex-specific beneficial effect of H_2_ was associated with estrogen and brain ER*β*-BDNF signaling in AD pathogenesis [135].

In clinical human research, a previous study has found that H_2_ administration did not change the Alzheimer's Disease Assessment Scale-cognitive subscale (ADAS-cog) scores after one year in patients with mild cognitive impairment. However, in the H_2_ group of apolipoprotein E4 genotype carriers, six and five out of seven subjects had improved ADAS-cog and word recall task scores [136].

#### 3.1.2. Effects of H_2_ on PD

In animal experiments, 6-hydroxydopamine (6-OHDA) and 1-methyl-4-phenyl-1,2,3,6-tetrahydropyrine (MPTP) are neurotoxic by generating ROS and are therefore often used to produce models of PD [137]. In a 6-OHDA-induced PD model, drinking 50% saturated HW before or after stereotactic surgery was found to prevent development and progression of the nigrostriatal degeneration, effectively preventing the dopaminergic neuron loss [138]. In MPTP-induced (including acute and chronic) PD, drinking HW significantly reduced the loss of dopaminergic neurons. This effect was independent of H_2_ concentration in water, such that H_2_ significantly decreased MPTP-induced accumulation of cellular 8-oxoguanine (marker of DNA damage) and 4-HNE (marker of lipid peroxidation) and reduced oxidative stress in the brain [139]. Photobiomodulation (PBM) is an effective method to alleviate PD symptoms by enhancing mitochondrial function and boosting ATP production, although it is often accompanied by increased ROS production. Concomitant treatment with H_2_ and PBM for a week significantly improved the Unified Parkinson's Disease Rating Scale (UPDRS) scores and eliminated the adverse effect of PBM [140]. Brenner et al. have found that PD may be caused by melanin in the substantia nigra, which fails to produce molecular H_2_ from water dissociation and subsequently cannot protect the brain from oxidative stress. Therefore, restoring melanin function or providing supplemental H_2_ might be a potential therapy for PD [141].

A randomized clinical pilot study and a later multicenter study showed that drinking HW improved the total UPDRS scores, while placebo worsened them [142, 143]. However, a pilot study carried out by the same team has revealed that the inhalation of molecular H_2_ gas was safe but did not show any beneficial effects in patients with PD [144]. Another study has shown that inhaling a 1.2–1.4% H_2_-air mixture for 10 min twice a day for four weeks did not significantly influence the clinical PD parameters but increased urinary 8-OHdG levels. Researchers explained that the increased ROS levels are not always associated with toxicity and disease. They also have essential roles in modulating the cellular adaptation process known as hormesis, which exerts a cytoprotective effect. This beneficial increase in oxidative stress effect of H_2_ is partly mediated by hormetic mechanisms [145].

### 3.2. Effects of H_2_ on CVDs

Ageing has a prominent effect on the cardiovascular system, leading to an increase in incidence of CVDs, such as atherosclerosis, myocardial infarction, hypertension, and stroke [146, 147]. H_2_ can protect the heart and blood vessels from ageing-related degeneration.

#### 3.2.1. Effect of H_2_ on the Heart

H_2_ can protect the heart from myocardial infarct injuries and alleviate cardiohypertrophy and heart failure. HRS significantly alleviated the inflammation and apoptosis induced by myocardial I/R injury by activating PINK1/Parkin-mediated mitophagy [116]. In a swine model, inhalation of 2% H_2_ gas improved myocardial stunning. When the inhalation concentration was increased to 4%, H_2_ gas significantly reduced myocardial infarct size [148]. In humans, oxidative stress and inflammation are the primary risk factors in hypertension-caused left ventricular hypertrophy [149–151]. Chronic treatment with HRS effectively attenuated left ventricular hypertrophy in rats, restored the activity of antioxidant enzymes, suppressed NADPH oxidase activity, inhibited NF-*κ*B activation and proinflammatory cytokines, and alleviated pressure overload-induced interstitial fibrosis and cardiac dysfunction in rats [38, 152]. H_2_ can especially alleviate mitochondrial dysfunction in hypertensive cardiac hypertrophy by restoring ETC enzyme activity and increasing levels of ATP production in the left ventricle [152].

In addition, H_2_ improved interstitial fibrosis in the heart. In pressure-overloaded heart injury, H_2_ suppressed TGF-*β*1 signaling, effectively preventing heart failure [38, 153]. Moreover, H_2_ inhibited p53-mediated apoptosis and alleviated progression of chronic heart failure [154].

So far, the evidence for the protective effect of H_2_ on the heart has been restricted to animal experiments and human studies remain limited. Interestingly, a prior study has found that a decrease in exhaled H_2_ during night sleep was associated with congestive heart failure (CHF) severity and can be used as a marker of CHF [155].

#### 3.2.2. Effect of H_2_ on Blood Vessels

The vasculature is composed of endothelial cells, vascular smooth muscle cells (VSMCs), and fibroblasts. These components influence each other in an autocrine or paracrine manner [156]. Vascular ageing is a progressive decline of vascular function, including endothelial dysfunction, inflammation, proliferation, fibrosis, and calcification in VSMCs [157, 158]. Therefore, it is one of the major risk factors of ageing-related CVDs.

HRW intake decreased serum concentrations of oxidized low-density lipoprotein (LDL) and free fatty acids and improved high-density lipoprotein (HDL) function and glucose metabolism [12, 159, 160]. In an apolipoprotein E knockout mouse model of spontaneous atherosclerosis development, drinking HW for four months significantly reduced atherosclerotic lesions and decreased oxidative stress level in the aorta [161]. H_2_ can also stimulate Sirt1-mediated autophagy and attenuate oxidized LDL-induced inflammation [64]. Treatment with HRS in hypertensive rats markedly alleviated vascular dysfunction, restored baroreflex function, and modulated NO bioavailability by abating oxidative stress, suppressing inflammation, and preserving mitochondrial function [152].

### 3.3. Effect of H_2_ on Ageing-Related Pulmonary Disease

COPD and idiopathic pulmonary fibrosis are regarded as lung diseases related to accelerated ageing, which exhibit all of the hallmarks of ageing [162]. COPD is the fourth leading cause of death in the world, with a particularly increasing prevalence in the elderly people [163]. It is an abnormal response to chronic inflammation and injury with excessive activation of macrophages, neutrophils, lymphocytes, and fibroblasts in the lungs, leading to breathlessness and reduction in exercise tolerance [164]. The etiology of COPD involves exposure to external noxious particles or gases, particularly during CS and indoor cooking [163]. Pulmonary fibrosis is one of the major causes of morbidity, and there is still no effective treatment to abate the aberrant repair [165]. Research evidence has shown that ROS and inflammation play a crucial role in inducing a fibrotic response in the lungs by modulating extracellular matrix deposition [166, 167].

#### 3.3.1. Effect of H_2_ on COPD

H_2_ therapy may be a novel and effective treatment for COPD [164] with anti-inflammatory, antioxidant, and antiapoptotic effects [168].

In animal experiments, HRS significantly alleviated CS exposure caused by COPD, alleviated small-airway remodeling and goblet-cell hyperplasia in the tracheal epithelium, and reduced the number of inflammatory cells in the bronchoalveolar lavage fluid (BALF) [169, 170]. In addition, HRW treatment significantly reduced the mean linear intercept, restored static lung compliance, decreased the levels of oxidative DNA damage and senescence markers, and attenuated emphysema [51].

In clinical studies, inhalation of 2.4% H_2_-containing steam mixed with gas for 45 min in patients with asthma and COPD significantly attenuated the inflammatory status in the airways [88]. Similarly, a recent randomized multicenter clinical trial showed that combination therapy of H_2_ and oxygen was superior compared to single oxygen therapy in improving symptoms in patients with acute exacerbation of COPD (AECOPD). As a result, breathlessness, cough, and sputum scale scores were improved in the combination group [171]. This may provide a feasible alternative emergency management strategy for patients with AECOPD.

#### 3.3.2. Effect of H_2_ on Pulmonary Fibrosis

In bleomycin-induced pulmonary fibrosis, H_2_ inhalation reduced the ROS content. It specifically inhibited TGF-*β*1, decreased the expression level of mesenchymal cell marker vimentin, and increased the expression level of the epithelial cell marker E-cadherin, therefore inhibiting bleomycin-induced epithelial-to-mesenchymal transition (EMT) [172]. In a rheumatoid arthritis- (RA-) associated interstitial lung disease model, H_2_ decreased the levels of proinflammatory factors, apoptosis, and extracellular matrix molecules associated with RA pathogenesis and fibrosis. It also ameliorated oxidative stress by decreasing serum levels of lipid peroxide and 8-OHdG-positive cell numbers and alleviating RA-associated lung fibrosis [173].

So far, human studies on the action of H_2_ in pulmonary fibrosis are still lacking.

### 3.4. Effect of H_2_ on Metabolic Diseases

Ageing is associated with body composition changes that cause glucose intolerance and increase the risk of diabetes mellitus (DM). The incidence of DM increases with age as the general population's life expectancy also increases [174]. Type 2 diabetes mellitus (T2DM) is characterized by insulin resistance, hyperglycemia, and relative impairment in insulin secretion. Both genetic and environmental factors, such as obesity and ageing, play key roles in its pathogenesis [175]. Long-term HW drinking significantly improved obesity, hyperglycemia, and plasma triglyceride levels in genetically diabetic male db/db mice. This effect of H_2_ on hyperglycemia was similar to a diet restriction. H_2_ improved the expression of hepatic fibroblast growth factor 21 (HFGF21), which has the function of enhancing fatty acid and glucose expenditure [176]. By reducing oxidative stress and enhancing the antioxidative system, H_2_ may improve insulin resistance and alleviate the symptoms of DM [177].

In patients with T2DM or impaired glucose tolerance, consuming pure HRS for 8 weeks significantly improved lipid and glucose metabolism [12]. Another study found that after a single dose of acarbose in patients with T2DM, H_2_ gas production was inversely associated with a reduction in the peripheral blood IL-1*β* mRNA level [178]. Therefore, H_2_ potentially inhibited the inflammatory process in T2DM.

### 3.5. Effects of H_2_ on Cancer

There is no doubt that there is a link between ageing and cancer, where the incidence of cancer increases with age [179]. Although the molecular mechanisms underlying the association of ageing and cancer remain unknown, increased ROS levels, products of oxidative stress and mitochondrial dysfunction that occur in ageing and ageing-related disorders, have also been found in cancer [179].

Studies on H_2_ as an anticancer therapy can be traced back to 1975, when a two-week hyperbaric administration of H_2_ gas caused a marked regression in skin tumors [8]. Since then, mounting evidence has shown that H_2_ has an anticancer effect in various types of cancer via diverse mechanisms.

By reducing hepatic oxidative stress, apoptosis, and inflammation, H_2_ prevents progression of nonalcoholic steatohepatitis-related hepatocarcinogenesis [180]. However, a previous study has found that combining H_2_ with platinum nanocolloids exerts carcinostatic and carcinocidal effects by increasing H_2_ peroxide generation and cell death in a human gastric cancer cell line NUGC-4 [181]. It can also be inferred that H_2_ had an enhancing ROS effect in cancer cells but protected normal cells by inhibiting ROS. By downregulating chromosome 3, which is a regulator of chromosome condensation, H_2_ inhibits lung cancer progression [127].

H_2_ can also enhance the anticancer effects when combined with other therapies. HW combined with 5-fluorouracil enhanced cell apoptosis in colon cancer cells [182]. A recent study has found that hydrogenated palladium nanocrystals used as multifunctional H_2_ carriers together with near-infrared irradiation caused a higher initial ROS loss, more apoptosis, and severe mitochondrial metabolism inhibition in cancer cells, significantly enhancing the anticancer efficacy of thermal therapy [183].

In addition, H_2_ can alleviate the side effects of other anticancer therapies, such as chemotherapy and radiotherapy, improving quality of life in cancer patients. For example, H_2_ protected irradiated cells from oxidative damage and consequent apoptosis by reducing oxidative stress and inflammation [184] and attenuated gefitinib-induced exacerbation of naphthalene-evoked acute lung injury while not impairing antitumor activity [185]. A previous study has found that intraperitoneal injection of HRS ameliorated mortality, cardiac dysfunction, and histopathological changes caused by doxorubicin in a rat model [186].

In patients with advanced non-small-cell lung cancer, two weeks of H_2_ inhalation can significantly reverse adaptive and innate immune system senescence [187]. H_2_ therapy can decrease tumor progression and alleviate the adverse events of medications [188]. In patients with advanced colorectal cancer, H_2_ restored the exhausted cluster of differentiated (CD)8+ T cells and improved prognosis [189].

H_2_ therapy in ageing-related diseases is summarized in Table 1.

## 4. Administration Routs of H_2_

H_2_ can be easily administered in multiple ways, including inhalation, injection of HRS, drinking HRW, and bathing in HW (Table 2). There are several factors that may limit the clinical use of H_2_. For example, H_2_ is considered unsafe at a concentration of 4%, which is explosive and might have cytotoxic effects. Inhalation of H_2_ achieves a slower increase in its concentration compared to other administration routes [190].

## 5. Conclusion and Perspectives

Although modern medicine has evolved rapidly in the 21^st^ century, many significant questions still need to be addressed and many diseases still cannot be cured. As a “philosophical molecule,” H_2_ may overcome intractable diseases and ageing [41] and solve various problems via its use alone or synergistically with other therapies. Moreover, H_2_ gas has demonstrated a safety profile in a number of research studies, which is pivotal for clinical trials. H_2_ modulates ageing mainly via antioxidative and anti-inflammatory effects. In addition, it can regulate autophagy, mTOR, mitochondria, and apoptosis. All of these factors contribute to the ageing process and may take part in ageing-related diseases. However, the details of specific molecular mechanisms for the antiageing H_2_ effects still need further investigation, especially because ageing is a complex and multifactor process. To date, nine ageing hallmarks have been identified. In addition to the hallmarks discussed above, the influence of H_2_ on other hallmarks needs further study. For example, proteostasis can be destroyed by ROS and lead to protein oxidation. Protein oxidation can be divided into reversible and irreversible modifications [191], in addition to counteracting protein damage by proteolysis and autophagy. Whether H_2_ can repair the reversible protein oxidation through its antioxidative effect is unclear. Stem cell exhaustion is another ageing hallmark. Different ROS doses have different roles in regulating stem cells. Low ROS levels are regulated by intrinsic factors (cell respiration or NADPH oxidase activity) and extrinsic factors (stem cell factors or prostaglandin E2) to maintain stem cell self-renewal. However, high ROS levels due to stress and inflammation may cause stem cell exhaustion, induce stem cell differentiation, and enhance motility [192]. Whether H_2_ can modulate and maintain ROS at a suitable level and facilitate stem cell metabolism requires further study. In addition to the nine hallmarks above, circadian clocks modulate various biological processes and are progressively lost during the ageing process. Disruption of the circadian clock may influence the ageing process and pathogenesis of age-related diseases. Progressive loss of the circadian clock is also categorized as the common hallmark of ageing [193]. Studies have found that there is a connection between the circadian clock and oxidative stress [194, 195]. Interestingly, intestinal microbiota that regularly produce H_2_ gas also undergo diurnal oscillations in function and composition, and the amount of H_2_ generated varies depending on the individual and time of day. Therefore, there may be some interconnectedness between H_2_ and circadian rhythms [190], and this mechanism still needs to be elucidated. In addition, recent investigations about reductive stress, the counterpart of oxidative stress, which is defined as a condition of excess accumulation of reducing equivalents [196], have shown that overexpression of antioxidant enzymatic systems can lead to excess reducing equivalents and deplete ROS. Furthermore, feedback regulation establishment in which chronic reductive stress induces oxidative stress, in turn stimulates reductive stress [197]. Whether a long-term H_2_ administration elicits reductive stress and influences ageing and ageing-related diseases requires further study in the future. Finally, many of the studies on H_2_ have been limited to the topics of ageing-related diseases and may not be directly related to ageing under normal physiological conditions. The majority of the studies on H_2_ have been performed using *in vivo* animal and *in vitro* cell models. Therefore, its applications in humans remain unknown and require clinical studies to validate. Therefore, further long-term studies are needed to investigate the influence of H_2_ on the process of physiological ageing. Nevertheless, we believe that H_2_ plays a critical role in the ageing process and ageing-related diseases, providing optimistic prospects for therapy in this area.

## Figures and Tables

**Table 1:** Mechanisms of H_2_ in multiple ageing-related diseases.

Diseases		Effect of H_2_	References (cell/animal/human)
Neurodegenerative diseases	Alzheimer's disease	Inhibits JNK, nuclear NF-*κ*B, IL-6, TNF-*α*, and IL-1*β*; inhibits MAD and 8-OHdG; upregulates Sirt1-FoxO3a; and ER*β*-BDNF signaling.	[132] Sprague-Dawley rats; [133] Sprague-Dawley male rats; [134] SK-N-MC cells; and [135] APPswe/PS1dE9 mice.
	Parkinson's disease	Prevents dopaminergic neuron loss; decreases 8-OHdG and 4-HNE; and hermetic regulation by increasing 8-OHdG.	[138] Sprague-Dawley rats; [139] C57BL/6J mice; and [145] human.
	Heart	Activates PINK1/Parkin-mediated mitophagy; restores ETC enzyme activity; increases ATP production; suppresses NADPH oxidase; inhibits NF-*κ*B; and inhibits p53-mediated apoptosis.	[38] Wistar rats; [116] Wistar rats and H9C2 cells; [152] spontaneously hypertensive rats and Wistar-Kyoto rats; and [154] Sprague-Dawley rats.
	Blood vessels	Decreases oxidized LDL; improves HDL function and glucose metabolism; activates Sirt1-mediated autophagy; and modulates NO bioavailability.	[12] human; [64] RAW264.7 cell; [152] spontaneously hypertensive rats and Wistar-Kyoto rats; [159] human; and [160] human.
	COPD	Alleviates small-airway remodeling and goblet-cell hyperplasia; restores static lung compliance; reduces inflammatory cells in BALF; and decreases oxidative DNA damage.	[51] senescence marker protein 30 knockout mice; [169] C57BL mice; and [170] Sprague-Dawley rats.
	Pulmonary fibrosis	Reduces ROS content; inhibits TGF-*β*1and EMT; increases E-cadherin; and decreases 8-OHdG-positive cell numbers.	[172] Wistar rats; [173] D1CC transgenic mice.
Metabolic diseases	DM	Improves obesity and lipid and glucose metabolism; improves insulin resistance; increases HFGF21; and inhibits peripheral blood IL-1*β* mRNA.	[12, 176, 177] human; [176] Sprague-Dawley rats, C57BL/6 mice, and db/db mice; and [177] Sprague-Dawley rats.
Cancer		Inhibits ROS, apoptosis, and inflammation in lesion tissue; downregulates chromosome 3; enhances anticancer effects; alleviates side effects of anticancer therapies; modulates immune function; and restores exhausted CD8+ T cells.	[127] A549 and H1975 cells; [180] C57BL/6 mice; [182] mouse colon carcinoma cell line and BALB/c mice; [185] C57BL/6J mice and human lung cancer cell lines A549; [186] Wistar albino rats; [187] human; and [189] human.

**Table 2:** Possible H_2_ administration routes and their characteristics.

Possible H_2_ administration routes	Advantages and issues
H_2_ inhalation	Simple and easy; rapid action (concentration below 4% to prevent risk of explosion)
Oral intake HW	Practical and safe (H_2_ must be stored in an aluminum container to avoid a decrease in H_2_ concentration)
Intravenous or intraperitoneal injection of HS	Allows for H_2_ delivery with great efficacy and highly accurate doses
H_2_ bathing	H_2_ can reach the entire body in only 10 min after bathing safely and easily

## Abbreviations

4-HNE: 4-Hydroxynonenal
6-OHDA: 6-Hydroxydopamine
8-OHdG: 8-Hydroxy-2′-deoxyguanosine
AD: Alzheimer's disease
ADAS-cog: Alzheimer's Disease Assessment Scale-cognitive subscale
AECOPD: Acute exacerbation of COPD
AMPK: AMP-activated protein kinase
Atg: Autophagy-related genes
ATP: Adenosine triphosphate
BALF: The bronchoalveolar lavage fluid
CAT: Catalase
CD: Cluster of differentiation
COPD: Chronic obstructive pulmonary disease
CS: Cigarette smoke
CHF: Congestive heart failure
CVD: Cardiovascular disease
DM: Diabetes mellitus
ETC: Electron transport chain
EMT: Epithelial-to-mesenchymal transition
FoxO3a: Forkhead box protein O3a
GPX1: Glutathione 1
HDL: High-density lipoprotein
HFGF2: Hepatic fibroblast growth factor 21
HO-1: Heme oxygenase-1
HRS: Hydrogen-rich saline
HRW: Drinking hydrogen-rich water
HS: Hydrogen saline
HW: Hydrogen water
I/R: Ischemia-reperfusion
IL: Interleukin
JNK: c-Jun NH₂-terminal kinase
LDL: Low-density lipoprotein
MPO: Myeloperoxidase
MPTP: Methyl-4-phenyl-1,2,3,6-tetrahydropyrine
mtDNA: Mitochondrial DNA
NADPH: Nicotinamide adenine dinucleotide phosphate
NF-*κ*B: Nuclear factor kappa B
Nrf2: NRF-E2-related factor2
PBM: Photobiomodulation
PD: Parkinson's disease
RA: Rheumatoid arthritis
RNS: Reactive nitrogen species
ROS: Reactive oxygen species
SOD: Superoxide dismutase
T2DM: Type 2 diabetes mellitus
TGF: Transforming growth factor
TLR: Toll-like receptors
TNF-*α*: Tumor necrosis factor-*α*
UPDRS: Unified Parkinson's Disease Rating Scale
VSMCs: Vascular smooth muscle cells.
